# Blood meal identification and parasite detection in laboratory-fed and field-captured *Lutzomyia longipalpis* by PCR using FTA databasing paper

**DOI:** 10.1016/j.actatropica.2008.06.003

**Published:** 2008-09

**Authors:** Mauricio R.V. Sant’Anna, Nathaniel G. Jones, Jonathan A. Hindley, Antonio F. Mendes-Sousa, Rod J. Dillon, Reginaldo R. Cavalcante, Bruce Alexander, Paul A. Bates

**Affiliations:** a Liverpool School of Tropical Medicine, Pembroke Place, Liverpool L3 5QA, UK; b Departamento de Parasitologia e Microbiologia, CCS, Universidade Federal do Piaui, Teresina, PI, Brazil

**Keywords:** FTA, Cytochrome *b*, Multiplex PCR, *Leishmania infantum*, *Lutzomyia longipalpis*, Blood meal

## Abstract

The phlebotomine sand fly *Lutzomyia longipalpis* takes blood from a variety of wild and domestic animals and transmits *Leishmania* (*Leishmania*) *infantum chagasi*, etiological agent of American visceral leishmaniasis. Blood meal identification in sand flies has depended largely on serological methods but a new protocol described here uses filter-based technology to stabilise and store blood meal DNA, allowing subsequent PCR identification of blood meal sources, as well as parasite detection, in blood-fed sand flies. This technique revealed that 53.6% of field-collected sand flies captured in the back yards of houses in Teresina (Brazil) had fed on chickens. The potential applications of this technique in epidemiological studies and strategic planning for leishmaniasis control programmes are discussed.

## 1 Introduction

Zoonotic visceral leishmaniasis (ZVL) represents a serious threat to public health in both the Old and New World. It is the most severe form of leishmaniasis, with fulminating disease usually being fatal if untreated and most prevalent in malnourished children (Cerf et al., 1987). About 90% of cases occur in economically deprived rural and suburban areas in three geographical regions: the Indian subcontinent, East Africa and South and Central America, with Brazil contributing most cases in the New World (Dantas-Torres and Brandão-Filho, 2006).

During the last 20 years ZVL due to *Leishmania* (*Leishmania*) *infantum chagasi* has become increasingly urbanised and is now present in several Brazilian cities (Michalsky et al., 2007). Although more common in socio-economically disadvantaged populations occupying the “vilas” or “favelas”, the juxtaposition of these areas with wealthy neighbourhoods means that ZVL is a potential threat to the whole population (Alexander et al., 2002). The sand fly *Lutzomyia longipalpis* (Lutz and Neiva, 1912) is the urban vector of *Le. infantum* in Brazil. Originally associated with open Savannah, it is able to colonize urban areas and other habitats affected by human activities, taking blood meals from a wide variety of hosts, including livestock, dogs and chickens (Lainson and Rangel, 2005). Widely differing observations have been made regarding the degree to which *Lu. longipalpis* bites humans in different habitats, indicating that this species has no strong innate host preference. The absence of a common term to distinguish *Lu. longipalpis* from other biting insects in urban ZVL foci suggests that this fly does not constitute a substantial biting nuisance for the inhabitants (Alexander et al., 2002).

Identification of the blood meals of haematophagous insects to date has largely depended on serological techniques such as the precipitin test, latex agglutination test and the enzyme-linked immunosorbent assay (ELISA) (Boorman et al., 1977; Washino and Tempelis, 1983; Beier et al., 1988; Gomes et al., 2001; Mwangangi et al., 2003). Although these methods have yielded important information on the identity of the vertebrate hosts of many blood-feeding arthropods, they are time-consuming and lack sensitivity. PCR-based identification of vertebrate host blood meals is a potentially convenient alternative, which has already been performed on several vectors including ticks (Pichon et al., 2003; Estrada-Peña et al., 2005), triatomine bugs (Bosseno et al., 2006; Pizarro et al., 2007) and mosquitoes. PCR based on primers designed from multiple alignments of the mitochondrial cytochrome *b* gene have identified avian and mammalian hosts of various species of mosquito (Ngo and Kramer, 2003; Kent and Norris, 2005; Molaei et al., 2006; Kent et al., 2006). PCR-RFLP cytochrome *b* analysis was also used to identify the origin of blood meals in the tick *Ixodes ricinus* (Kirstein and Gray, 1996) and tsetse flies (Steuber et al., 2005).

Until recently sand fly host identification by blood meal analysis has been limited to serological studies using ELISA (Gomez et al., 1998; Agrela et al., 2002; Bongiorno et al., 2003; Svobodová et al., 2003; Marassá et al., 2006; Rossi et al., 2008), counter immunoelectrophoresis (Morsy et al., 1993), agarose gel diffusion (Srinivasan and Panicker, 1992), precipitin test (Tesh et al., 1971, 1972; Javadian et al., 1977; Morrison et al., 1993; Nery et al., 2004; Afonso et al., 2005) and a more laborious histological technique (Guzman et al., 1994). The first PCR-based method using the prepronociceptin gene has been recently described (Haouas et al., 2007). One of the major constraints in developing a successful PCR-based method for sand flies is that they are diminutive insects, only able to ingest very small quantities of blood (Rogers et al., 2002). Template stability and the lack of uniformity in DNA template concentration are also a concern, particularly as template degradation during blood digestion has been observed for mosquitoes (Mukabana et al., 2002; Ngo and Kramer, 2003; Kent and Norris, 2005). However, preservation of specimens under field conditions coupled with the need for a rapid and sensitive method to identify sand fly hosts is important for the study of vector–vertebrate host associations, as well as to improve current control interventions targeting *Lu. longipalpis*.

The FTA filter methodology has been shown to be a useful tool in the storage and PCR detection of DNA in a wide variety of organisms, including trypanosome identification in wild tsetse populations (Adams et al., 2006), diagnosis and surveillance of *Trypanosoma vivax* in ruminants (Gonzales et al., 2006), PCR detection of *Cyclospora cayetanensis* and *Cryptosporidium parvum* from food samples and human faecal specimens (Orlandi and Lampel, 2000), as well as archiving and processing DNA from fresh water protozoans (Hide et al., 2003) and extraction and storage of insect DNA in forensic entomology (Harvey, 2005). In the present study, we adapted a multiplex PCR protocol (Kent and Norris, 2005; Ngo and Kramer, 2003) and combined this with a FTA-based technology to store and analyse sand fly samples from the field, in order to identify putative hosts of this important disease vector in urban habitats.

## 2 Materials and methods

### 2.1 Laboratory-reared insects

A laboratory colony of *Lu. longipalpis* established from sand flies caught in Jacobina (Bahia, Brazil) and kept at the Liverpool School of Tropical Medicine was used in the blood-feeding time course experiments and maintained using standard methods (Modi, 1997). Insects were reared under controlled conditions of temperature (28 ± 1 °C) and humidity (80–95%) and the adult female insects were fed on hamsters twice a week. All procedures involving animals were approved by a local Animal Welfare Committee and performed in accordance with UK Government (Home Office) and EC regulations. Insects were provided with 70% sucrose for 96 h post-emergence until they were blood-fed for DNA degradation experiments.

### 2.2 Study area, sand fly capture and handling

Field specimens of *Lu. longipalpis* were collected by hanging CDC light traps (Alexander, 2000) from 18:00 to 08:00 h for a total of 65 trap-nights during May–June 2007 in houses of two neighbourhoods (Satélite and Ininga) of Teresina (5°5′20″S and 42°48′07″W), capital of the Brazilian state of Piauí. Active transmission of both canine and human ZVL occurs in this city (Werneck et al., 2007). Twenty-four traps were set up in Ininga, a relatively high-income neighbourhood near the University of Piauí, whereas 41 were placed in Satélite, a lower income neighbourhood. Live insects were collected in the morning following capture and transported to the lab within 2 h. They were then identified according to Young and Duncan (1994), and were either immediately frozen at −20 °C for subsequent whole DNA extraction or homogenised in 0.15 M saline using a cordless motorized pestle and spotted onto Whatman^®^ FTA cards for DNA storage/extraction.

### 2.3 Genomic DNA isolation

Whole genomic DNA was extracted from laboratory-fed single sand flies using the FastDNA SPIN Kit for Soil (Q BIOgene). The manufacturer's protocol was modified and optimized for sand fly genomic DNA extraction. Briefly, 20 human blood-fed and 30 chicken blood-fed sand flies were homogenised individually in 1.5 mL microcentrifuge tubes with 244.5 μL sodium phosphate buffer and 30.5 μL MT buffer (both provided with the kit) using a cordless pestle pellet motor on ice. After centrifugation at 14,000 × *g* for 8 min, the supernatant liquid was transferred to a clean tube, followed by the addition of 65 μL of protein precipitation solution (PPS). After another centrifugation step (14,000 × *g* for 7 min), the supernatant was transferred to another tube and mixed with 0.5 mL of binding matrix. Tubes were then placed in a rotator for 2 min to agitate the mixture gently and allow DNA binding with the matrix, and subsequently placed in a rack for 3 min for silica matrix settling. A 250 μL fraction of the supernatant was discarded and the remainder used to resuspend the matrix. The mixture was then applied to a SPIN filter cartridge and centrifuged at 14,000 × *g* for 1 min. Filters were then washed with 250 μL salt/ethanol wash solution (SEWS-M) and centrifuged at 14,000 × *g* for 1 min. After an extra centrifugation step to dry the matrix of residual ethanol, genomic DNA was eluted with 50 μL of RNase-free water after centrifugation at full speed for 1 min. DNA yield was quantified using a Nanodrop ND-1000 Spectrophotometer and approximately 10 ng DNA template for each sample was used in a multiplexed PCR reaction and visualised in 1.5% agarose/ethidium bromide gels.

### 2.4 Template preparation on FTA filter paper

Forty seven human blood-fed, 49 chicken blood-fed and 58 wild-caught blood-engorged sand flies were homogenised individually in 50 μL of 0.15 M saline using a cordless motorized pestle and the whole volume spotted onto a Whatman^®^ FTA card. The homogenates were allowed to air-dry and the FTA cards kept in sealed plastic bags at room temperature until required. For PCR, discs of diameter 2 mm were punched out and placed in a 1.5 mL microcentrifuge tube. FTA disks were washed three times with 0.5 mL of FTA purification buffer for 5 min, twice with 0.2 mL of 10 mM Tris (pH 8.0) containing 0.1 mM EDTA for 5 min at room temperature and then air-dried on a heating block at 56 °C for 10 min. These washed filters were used directly in a multiplexed PCR reaction and visualised in 1.5% agarose/ethidium bromide gels.

### 2.5 PCR primers and reaction conditions

PCR primers based upon alignment of cytochrome *b* sequences of mammalian and avian species were obtained from previously published primer sequences (Table 1). The multiplexed PCR was optimized for sand fly templates. An initial denaturation of 3 min at 94 °C was followed by 35 cycles at 94 °C for 1 min, 52 °C for 1 min and 72 °C for 1 min. The final extension step was 72 °C for 10 min. Field-captured engorged and unfed sand flies were also screened for *Leishmania* infection in a separate PCR reaction using previously published primer sequences, targeting either the kDNA or the small subunit of the rRNA gene (Lachaud et al., 2002).

### 2.6 Blood sample source and blood feeding

Chicken blood in Alsevers anticoagulant was purchased from TCS Biosciences and human blood obtained from the National Blood Service (Speke, Liverpool, UK). For time-course experiments, sand flies were fed on chicken and human blood through chick-skin feeders at 35 °C and each group sampled at 24, 48, 72, 96, and 120 h intervals from the day of the feed (day 0). Sand flies were either frozen at −20 °C for genomic DNA extraction or homogenised in 50 μL of 0.15 M saline and spotted onto Whatman^®^ FTA cards for DNA extraction as above.

### 2.7 Parasite preparation and sand fly infection with *Le. infantum*

Frozen aliquots of *Le. infantum* amastigotes obtained from spleen homogenates of infected female BALB/c mice were rapidly thawed from liquid nitrogen and gently mixed with 5 mL complete M199 medium (Sigma). Parasites were centrifuged at 1500 × *g* for 10 min and washed with 5 mL M199 medium twice, before being re-suspended in 10 mL of complete M199 medium containing 10% FBS and transferred to a culture flask and cultured at 26 °C for 48 h. In preparation for infection, 2 mL of heat-inactivated rabbit blood was used to re-suspend cultured parasites to a final concentration of 1 × 10^6^ parasites/mL. Rabbit blood seeded with parasites was offered to 5-day-old *Lu. longipalpis* through a chick-skin membrane and infected female sand flies collected daily from 0 to 5 days post-feeding.

### 2.8 Parasite detection in lab-infected *Lu. longipalpis* with *Le. infantum* using FTA extraction

Infected sand flies were homogenised in 50 μL of 0.15 M saline and spotted onto Whatman^®^ FTA cards. Discs were extracted as described above and part of the parasite small subunit ribosomal RNA (SSU rRNA) was amplified by PCR using the genus-specific primers R221 and R332 as described in Lachaud et al. (2002). To determine the sensitivity of parasite detection, 5 × 10^4^ of cultured *Le. infantum* parasites were serially diluted and spotted onto Whatman^®^ FTA cards for DNA extraction and the parasite SSU rRNA detected as above.

## 3 Results

**Fig. 1:**
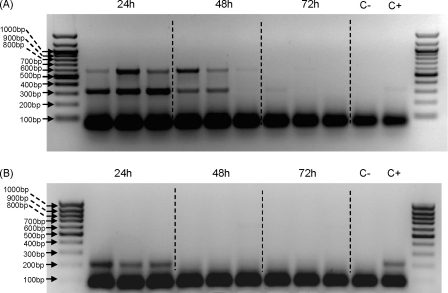
Time course analysis of genomic DNA extracts for sand flies fed on human and chicken blood at 24, 48, and 72 h after the blood feed. Genomic DNA extractions were followed by multiplex PCR reactions using primer sequences for human, dog, chicken, universal forward and reverse for the cytochrome *b* gene. (A) Sand flies fed on human blood. (B) Sand flies fed on chicken blood. External lanes correspond to 100 base pair (bp) DNA ladder. C− and C+ are negative (no DNA) and positive control reactions, respectively.

**Fig. 2:**
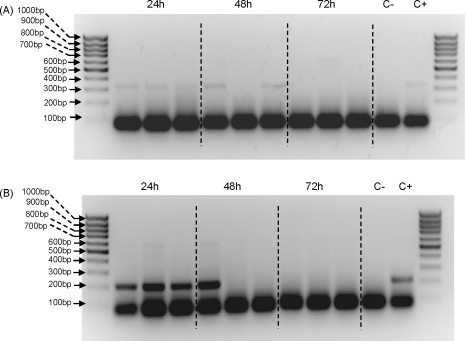
Time course analysis of FTA extractions for sand flies fed on human and chicken blood at 24, 48 and 72 h after the blood feed. FTA extractions were followed by multiplex PCR reactions using primer sequences for human, dog, chicken, universal forward and reverse for the cytochrome *b* gene. (A) Sand flies fed on human blood. (B) Sand flies fed on chicken blood. External lanes correspond to 100 bp DNA ladder. C− and C+ are negative (no DNA) and positive control reactions, respectively.

To optimise PCR conditions initial experiments were performed on DNA isolated from sand flies using a genomic DNA extraction kit. Time-course analysis on chicken and human-fed sand flies in the laboratory showed that host DNA could be detected for up to 48 h after the blood meal (Fig. 1). Prominent non-specific low-molecular weight products were observed in all lanes, including control reactions lacking DNA, and are often seen in multiplex PCR reactions. However, with human DNA two specific bands were detected, corresponding to the predicted human-specific 334 bp and universal mammalian 623 bp bands (Fig. 1A). The lack of products in samples taken after 48 h is presumably due to digestion of the DNA by sand fly gut enzymes. Using flies fed on chicken blood the predicted 210 bp band was observed (Fig. 1B). Next the same procedure was repeated, except that sand fly samples were homogenised and spotted onto FTA cards, then discs punched out and used in the multiplex PCR (Fig. 2). As before, specific bands were detected in sand flies up to 48 h after feeding on either human or chicken blood. Although the results were broadly similar, in comparison to directly extracted genomic DNA (Fig. 1), the human-specific bands were consistently weaker, whereas the chicken-specific bands were consistently stronger. In both sets of experiments the absence of specific bands after 48 h corresponds to the end of blood meal digestion, indicating likely template degradation by digestive enzymes.

**Fig. 3:**
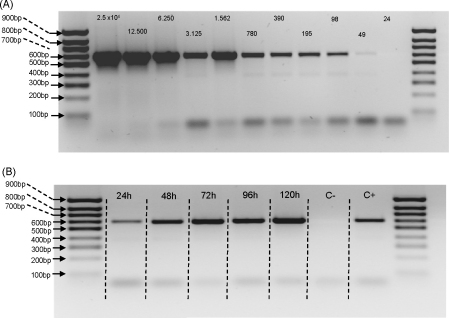
Detection of *Leishmania infantum* DNA using the FTA method. (A) Serial dilutions of cultured *L. infantum* were spotted onto FTA paper, then extraction and PCR performed using primers specific for the parasite small subunit ribosomal RNA. Numbers above bands represent the number of parasites spotted onto the FTA paper. (B) Detection of *L. infantum* in lab-infected *Lu. longipalpis* submitted to whole fly FTA extractions from 24 h to 120 h post-infection. Negative control (C−) represents FTA extraction from an uninfected sand fly, and positive control (C+) represents genomic *Leishmania* DNA extracted from an infected sand fly amplified using the same set of primers.

In addition to blood meal identification, it would also be useful to detect the presence of *Leishmania* parasites in sand fly homogenates. Therefore, a sensitivity test was performed by preparing parasite serial dilutions, which were then spotted onto FTA paper and processed for PCR using primers targeting the parasite small subunit ribosomal RNA (Lachaud et al., 2002) (Fig. 3A). The predicted 603 bp product was detected, and this showed that the equivalent of as few as 49 parasites/original 50 μl sample could be detected. Given that the assay uses a 2 mm disc from a spot of approximately 15 mm diameter this represents a theoretical sensitivity of approximately a single parasite (∼77 fg DNA). To assess whether *Leishmania* infection could be detected in samples of lab-reared *Lu. longipalpis*, flies were experimentally infected with *Le. infantum*, homogenised individually in 0.15 M saline and spotted onto FTA filter paper. A time course analysis of individual sand flies homogenised at various time points post-infection was able to detect the presence of parasite DNA as early as 24 h (Fig. 3B). As expected, the band intensity increased with time as the infection matured. Nevertheless, the results show that sand flies sampled 24–48 h after feeding could be analysed for both blood meal identification and the presence of parasites.

**Fig. 4:**
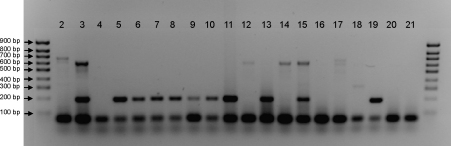
Agarose gel showing host-specific cytochrome *b* PCR products amplified from FTA extractions of blood-engorged wild caught sand flies. Lanes 2–12, sand flies captured in different houses, each lane representing a single sand fly captured in a single house; lanes 13–16, sand flies captured in a chicken house; lane 17, dog positive control; lane 18, human positive control; lane 19, chicken positive control; lanes 20 and 21, negative controls. Outside lanes contain a 100 bp ladder.

Finally the FTA method was tested on wild-caught sand flies collected in Teresina, Brazil. A total of 2089 sand flies (1701 males and 392 females) were recovered from 65 CDC traps set up in 61 houses from two distinct neighbourhoods during May–June 2007. Of these, 1739 were identified according to Young and Duncan (1994), 1732 as *Lu. longipalpis* (99.5%), four as *Lu. lenti* (0.23%) and one each (0.06%) as *Lu. whitmani*, *Lu. termitophyla* and *Lu. aragaoi*. All 58 blood-engorged female sand flies were *Lu*. *longipalpis* and positive blood meal identifications were obtained for 43 of these (74%). An example of the results obtained using the FTA extraction diagnostic assay on wild-caught sand flies is shown in Fig. 4. Sand flies that had fed on dogs (e.g. lane 2) and chickens (e.g. lanes 5–11 and 13) were detected. Some flies did not yield any PCR products (e.g. lanes 4 and 16), whereas others amplified the 623 bp universal band (e.g. lanes 3, 12, 14 and 15). In the example shown, two of these (lanes 3 and 15) also included the 210 bp chicken-specific band and were therefore included as chicken-fed sand flies in the overall analysis. Others (e.g. lanes 12 and 14) did not reveal any specific bands and were recorded as unidentified. In total, 41 (70.7%) of the 58 blood-engorged sand flies collected had fed on chickens, 2 (3.4%) had fed on dogs, and 15 (25.9%) could not be identified. These negative results could be due to loss of DNA during blood meal digestion, the presence of a small blood meal or feeding on a host not covered by the multiplex PCR *i.e*., non-chicken, dog or human blood meals. Fifteen (53.6%) of 28 engorged sand flies collected outside henhouses were positive for chicken blood, compared to 26/30 (86.7%) of specimens collected inside these shelters. None of the blood-fed sand flies collected seemed to have fed on human hosts. The absence of two specific bands in the PCR reactions suggests that none of the engorged sand flies contained blood from more than one host species. Finally, 205/392 field-collected female sand flies (52.3%) were screened for *Le. infantum* infection using two different set of primers to amplify fragments of the parasite small subunit ribosomal RNA (SSU rRNA) or kDNA (Lachaud et al., 2002). Parasite DNA could not be detected in any of the wild-caught sand flies analysed, indicating that the infection rate in this sample was less than 0.49% (1 in 205).

## 4 Discussion

We have adapted a PCR methodology first developed for mosquitoes (Kent and Norris, 2005; Ngo and Kramer, 2003) to identify blood meals from wild-collected sand flies in an urban area of *Le. infantum* transmission. This methodology was further developed for host identification by DNA immobilisation using FTA cards for long-term storage and subsequent PCR. Our results compare favourably with those recently described by Haouas et al. (2007), where genomic DNA was extracted from sand flies using a QiaAmp blood DNA mini Kit. In our study successful amplification was obtained when blood-engorged sand flies were homogenised in saline and spotted onto FTA databasing filter paper. FTA paper has a matrix designed to lyse cells upon contact and sequester DNA within the paper matrix (Smith and Burgoyne, 2004). FTA filters also eliminate laborious isolation and purification steps, preserving DNA integrity and eliminating potential sources of target DNA losses through degradative processes associated with conventional methodologies (Orlandi and Lampel, 2000). Moreover, the easy handling and long-term stability of DNA blood samples stored on FTA filter papers makes this methodology robust and ideal in field conditions, where samples are often collected far away from where they are processed. It also provides a convenient and safe way to send samples between laboratories without a cold chain or transport of liquids. As shown in Figs. 1 and 2, FTA extractions were as effective as the genomic DNA extractions currently used from frozen/dried field samples. This method will be extremely useful for entomologically based projects involving blood-feeding behaviour and vectorial capacity of sand flies in urban and rural endemic areas of VL.

The time-course experiments showed that host DNA was detectable in chicken and human-fed control sand flies up to 48 h after the blood meal (Figs. 1 and 2). Similar results were obtained by Haouas et al. (2007) in their study of *Phlebotomus* species caught in central Tunisia and for *Anopheles* species captured in the wild (Kent and Norris, 2005). Boayke et al. (1999) and Ngo and Kramer (2003) detected human blood meals in black flies and avian blood meals in *Culex pipiens*, respectively, up to 72 h post-blood feeding. Although sand flies recently fed on chickens contain nearly double their normal DNA content compared to human-fed sand flies due to the nucleated avian erythrocytes (unpublished data), chicken blood meals could not be identified after 48 h (Figs. 1B and 2B). This represents one of the major constraints of any methodology based on blood identification within the insect's midgut. Due to blood digestion process inherent to any blood-sucking insect, only recently engorged sand flies may be used for the analysis.

The origin of blood meals in 15/58 of field-caught sand flies could not be identified (including four collected inside a henhouse). For those field specimens where vertebrate host DNA identification failed (Fig. 4), host DNA might have been insufficient for amplification, the process of digestion may have denatured the DNA or the sand fly may have fed on an animal not included in the screening. In some of our field specimens that were captured inside a henhouse, sand flies might have fed on different hosts and used the shelter as a resting site (Brazil et al., 1991). It is also worthwhile mentioning that the control band of 623 bp (common to all species in this study), derived from PCR reactions in which universal forward and reverse primers for the cytochrome *b* gene were used (Kent and Norris, 2005), could not be visualised in some of the PCR reactions. This was probably due to partial DNA template degradation in the midgut where one of the universal primers would anneal, probably accounting for reactions which only host-specific cytochrome *b* bands could be visualised. Sand flies ingest minuscule amounts of blood (Rogers et al., 2002) and there may be insufficient host DNA, especially in partially fed sand flies. Mukabana et al. (2002) showed that blood meals of *Anopheles gambiae* contained 2–82 ng of human DNA. More recently, Kent and Norris (2005) showed that at least 50 ng was necessary for visible amplification from mosquito abdomens. In our PCR amplifications, as little as 10 ng was sufficient for band visualisation using genomic DNA extractions (data not shown). Sand flies seem to concentrate their blood meals through prediuresis (Sádlová et al., 1998; Sádlová and Volf, 1999), including *Lu. longipalpis* (R. Dillon, unpublished observation) excreting fluid to concentrate proteins of the blood meals in a similar way to mosquitoes. This phenomenon is helpful, as it will increase the concentration of host DNA in the midgut and the probability of obtaining a good-quality template for PCR amplification.

Recently, PCR-based approaches have allowed diagnosis of infectious diseases, including *Leishmania* detection in human patients (Dweik et al., 2007; Kumar et al., 2007; Foulet et al., 2007), infected dogs (Solano-Gallego et al., 2007; Gomes et al., 2007; de Andrade et al., 2006) and phlebotomine sand flies (Paiva et al., 2006; Cabrera et al., 2002; Myskova et al., 2008; Ranasinghe et al., 2008). Laboratory infections of *Lu. longipalpis* with *Le. infantum* followed by whole sand fly homogenisation in Whatman filter paper (Fig. 3) showed that FTA technology can be also used for parasite DNA storage and parasite detection by PCR. Parasite DNA was successfully amplified using previously described genus-specific primers (Lachaud et al., 2002). This simple methodology could be very useful in epidemiological studies in endemic areas for leishmaniasis as specimens suspected to contain parasites could be easily stored and amplified for parasite detection/identification. In addition, safe parasite storage can be guaranteed as the reagents on the FTA paper are designed to kill pathogens upon contact and the papers protect DNA within the samples for several years at ambient conditions (Smith and Burgoyne, 2004).

Previous studies of *Lu. longipalpis*, for example in an endemic area of visceral leishmaniasis transmission in Colombia, have suggested this sand fly is an opportunistic feeder and is not highly anthropophilic nor strongly attracted to dogs (Morrison et al., 1993). In the same study it was reported that *Lu. longipalpis* preferred cows and pigs over chickens. This may be true in a rural environment where large domestic mammalian species occur in abundance, reflected in a reduced *Lu. longipalpis* vectorial capacity (Morrison et al., 1993). In the current study, 30/58 of the engorged sand flies were collected inside henhouses and the remaining 28 outside these structures. Fifteen of the latter insects had clearly fed on an avian host, most probably chickens (53.6%). This result is similar to previous studies on blood meal preference of *Phlebotomus papatasi* in an Iranian village where a precipitin test showed that over 57% of the sand flies fed on birds, mainly chickens and pigeons (Javadian et al., 1977). Furthermore *Lu. longipalpis* populations are maintained artificially high in Lapinha cave near Belo Horizonte, Brazil by the constant presence of live chickens (Lane, 1986). Henhouses seem to form a suitable man-made refuge for sand flies. A high proportion of residents in low-income neighbourhoods where VL is present keep chickens for a wide variety of reasons and this may be an important factor in the urbanisation of the disease, with both positive and negative effects of *Le. infantum* transmission (Alexander et al., 2002). Regarding positive effects, *Lu. longipalpis* readily feeds on chickens (Lainson, 1989) and large numbers can be collected from chicken houses (Genaro et al., 1990), suggesting that chickens may promote *Le. infantum* transmission by sustaining a large vector population near human dwellings. Various epidemiological studies have suggested that proximity to chickens is a risk factor for acquiring human (Corredor et al., 1989; Castellon and Domingos, 1991; Arias et al., 1996; Caldas et al., 2002) or canine VL (Moreira et al., 2003), although results have been mixed. Caldas et al. (2002) found that in some analyses chicken rearing was a VL risk factor but in others it appeared to exert a protective effect. This contradiction is unlikely to be resolved by epidemiological approaches alone.

Understanding the role of chicken rearing in the *Le. infantum* transmission cycle is important because domestic chickens are frequently kept by the urban poor, who are already at increased risk of VL. Therefore, the outcome of studies aiming to comprehend the vector/chicken host interactions could ultimately lead to a better applicability of control methods by health authorities in endemic areas under intense *Leishmania* transmission, perhaps suggesting removal of chicken coops from near human dwellings, banning chicken rearing from urban environments or even focal treatment of chicken coops (Alexander et al., 2002). Moreover, blood meal identification in field-caught sand flies can confirm a strong association between sand flies and chicken hosts in urban areas and also help to improve understanding the role of domestic chickens in transmission of *Le. infantum* in urban foci. Any attempt to determine the relative importance of blood meal sources may be biased by differences in the post-feeding accessibilities of insects that bit particular host species. The greater sensitivity of the method reported here means that information can be obtained from specimens that have ingested relatively small amounts of blood and may have moved some distance from the animals on which they fed.

## Figures and Tables

**Table 1:** Primer sequences, genes targeted and length of amplification products for the PCR blood meal identification and parasite detection assays

Primer	5′–3′ sequences	Target	Product size
Human741F	ggcttacttctcttcattctctcct^a^	Mitochondrial cytochrome *b* gene	334 bp
Dog368F	ggaattgtactattattcgcaaccat^a^	Mitochondrial cytochrome *b* gene	680 bp
UNFOR403	tgaggacaaatatcattctgagg^a^	Mitochondrial cytochrome *b* gene	623 bp
UNREV1025	ggttgtcctccaattcatgtta^a^	Mitochondrial cytochrome *b* gene	–
Galliforme forward	atttcggctccctattagcag^b^	Mitochondrial cytochrome *b* gene	210 bp
Galliforme reverse	gtccgatgtgaaggaagatacagatgaagaagaa^b^	Mitochondrial cytochrome *b* gene	–
R221	ggttcctttcctgatttacg^c^	small subunit ribosomal RNA (SSU rRNA)	603 bp
R332	ggccggtaaaggccgaatag^c^	small subunit ribosomal RNA (SSU rRNA)	–
KI3A	ccagtttcccgccccg^c^	kDNA^d^ minicircle (10,000 copies)	120 bp
K13B	ggggttggtgtaaaatagggc^c^	kDNA^d^ minicircle (10,000 copies)	–

a See Kent and Norris (2005).

b See Ngo and Kramer (2003).

c See Lachaud et al. (2002).

d Kinetoplast DNA.
